# Exploring Inequalities in the Use, Quality, and Outcome of the Diabetes Management Program of Indonesian National Health Insurance

**DOI:** 10.1089/heq.2023.0025

**Published:** 2023-09-27

**Authors:** Joko Mulyanto, Yudhi Wibowo, Dwi Arini Ernawati, Diyah Woro Dwi Lestari, Dionne S. Kringos

**Affiliations:** ^1^ Department of Public Health and Community Medicine, Faculty of Medicine, Universitas Jenderal Soedirman, Purwokerto, Indonesia.; ^2^ Department of Public and Occupational Health, Amsterdam University Medical Centres, University of Amsterdam, Amsterdam, The Netherlands.; ^3^ Department of Bioethics, Faculty of Medicine, Universitas Jenderal Soedirman, Purwokerto, Indonesia.; ^4^ Amsterdam Public Health Research Institute, Quality of Care, Amsterdam, The Netherlands.

**Keywords:** diabetes mellitus, inequalities, Indonesia, glycemic control, disease management, National Health Insurance

## Abstract

**Introduction:**

Access to diabetes management programs is crucial to control the increasing contribution of diabetes to the global burden of disease. However, evidence regarding whether such services are equally accessible for all population groups is still lacking, particularly in the context of low-middle-income countries and under the National Health Insurance (NHI). This study aimed to assess the extent of socioeconomic and geographical inequalities in the use, quality, and outcome of a diabetes management program for beneficiaries of Indonesian NHI.

**Methods:**

A total of 628 participants in the NHI diabetes management program in Banyumas District, Indonesia, were included in 2021 in this cross-sectional study. The main variables measured were regular visits to primary care facilities, standard medication, and glycemic control. The rate difference and rate ratio of age-sex standardized prevalence rates, as well as multiple logistic regressions, were used to measure the extent of inequalities.

**Results:**

Around 70% of participants regularly visited primary care facilities and received standard medication, but only 35% had good glycemic control. Highly educated participants were more likely to have regular visits compared to low-educated participants (odds ratio [OR] 1.92; 95% confidence interval [95% CI]: 1.04–3.56). Based on employment and type of NHI beneficiaries, a small extent and even reverse inequalities were found although these findings were insignificant statistically. Urban residents were also more likely to have regular visits (OR 6.61; 95% CI: 2.90–15.08), receive standard medication (OR 9.73; 95% CI: 3.66–25.90), and have good glycemic control (OR 3.85; 95% CI: 1.68–8.83) compared to rural residents.

**Conclusions:**

Evidence on the extent of socioeconomic inequalities is inconclusive but substantial geographical inequalities in the use, quality, and outcome of diabetes management programs exist among Indonesian NHI beneficiaries. Future implementation policies of the program should consider particularly the geographical characteristics of participants to avoid and reduce inequalities and, hence, the disease burden of diabetes.

## Introduction

The share of noncommunicable diseases (NCDs) in the global burden of disease, particularly in low-middle-income countries (LMICs), is increasing. According to the Global Burden of Disease 2019 study, five major NCDs, including diabetes, contributed to 36% of global morbidity and mortality.^1^ Approximately 10% of the global adult population has diabetes, and 75% of diabetic individuals are found in LMICs such as Indonesia.^2–4^ In Indonesia, the burden of diabetes increased by 50% from 2009 to 2019. The estimated prevalence of diabetes among adults in Indonesia in 2021 was around 10.6% (∼19.4 million individuals), and diabetes contributes to 6.5% of all-cause mortality in Indonesia.^5^ The rapid increase in diabetes prevalence has also increased the economic burden of diabetes, with 0.8% of Indonesia's gross domestic product being used to cover the medical costs of diabetes and its complications.^6^

One of the effective interventions for reducing the health impact of diabetes is a diabetes management program.^7^ A systematic review of studies conducted in 15 LMICs showed that these programs are effective in reducing diabetes-related morbidity and mortality.^8^ However, the effectiveness of such programs depends on people's access to these services. Studies in high-income countries and LMICs consistently show that vulnerable groups, such as those with low socioeconomic status, have low access to diabetes management programs, which may reduce the effectiveness of the program.^9^ Financial barriers have been identified as a reason for low access to diabetes management programs among vulnerable groups.^10^

The inclusion of a diabetes management program among the benefits covered by National Health Insurance (NHI) is intended to address financial barriers to accessing diabetes management services. However, previous studies have shown that health care access is not solely determined by health insurance coverage, particularly for vulnerable groups.^11^ Although medical costs may be covered by health insurance, vulnerable people who need health services may still be hindered by indirect costs, such as transportation costs.^12^ Factors such as knowledge and cultural beliefs, personal experiences, health care trust, community dynamics, and information sources can also impact people's access to health services.^13^

Vulnerable groups often have low health literacy and are more susceptible to negative health care experiences which may hinder those individuals from understanding the benefit of specific health care such as diabetes management programs, impact their trust in health authorities, and lead to low utilization of such health care.^14^ Moreover, cultural factors such as norms, beliefs, and trust play a critical role in the decision to use health care, and these factors tend to influence vulnerable groups strongly which may cause the low use of health care for this particular group.^15,16^ In the context of Indonesia, studies have shown that poorer groups use health services less frequently than wealthier groups, even though all of these groups are covered by the NHI program.^17^ This suggests the potential for inequalities in access to diabetes management programs among NHI beneficiaries with different backgrounds.

Inequalities in access to diabetes management programs can have a significant impact on inequity in diabetes prevalence and lead to health inequity in general. Health equity refers to the principle that all individuals should have the opportunity to attain their highest level of health regardless of their social or economic circumstances. A previous study showed that disparities in global diabetes prevalence with LMICs have higher prevalence compared to high-income countries. Conventional risk factors (e.g., family history of diabetes and obesity) cannot explain this finding which implies that other factors such as social determinants of health, including access to health care, may contribute to these inequalities.^18^ Addressing the underlying determinants of inequalities in diabetes prevalence, including access to diabetes management programs, becomes a crucial step to reduce the inequality in diabetes prevalence and health inequity in general considering that these determinants directly contribute to health inequity.

The diabetes management program under the Indonesian NHI has been in place since 2014. The program includes comprehensive health care for diabetes, including health promotion, preventive care, curative treatment, and rehabilitation.^19^ Approximately 83% of the Indonesian population (224 million people) is covered by the NHI, highlighting the importance of this program in controlling the growing burden of diabetes in Indonesia.^20^

Assessing equal access to the diabetes management program is crucial for understanding its effectiveness in reducing the burden of diabetes. However, empirical evidence in this field is still scarce, particularly in the context of LMICs. Most studies on access to diabetes management programs in LMICs have focused on facilitators and barriers to the use of these programs and clinical outcomes such as disease control.^21^ Furthermore, studies on inequalities in the use, quality, and outcome of diabetes management program, which were conducted among beneficiaries of NHI in the context of LMICs, are still lacking. Finding from this study can be used as the basis of evidence-based policies to reduce inequalities in the use, quality, and outcome of diabetes management program as part of reducing inequity in diabetes prevalence, as well as overall health inequity.

For this study, we selected samples from beneficiaries of the Indonesian NHI who were enrolled in the diabetes management program in Banyumas District, one of the largest districts in Central Java Province, Indonesia. Banyumas District has 1.8 million population, with an estimated diabetes prevalence of 1.7%, and the coverage of NHI is about 79% of the population. The situation of Banyumas District resembles most of the districts in Java and Sumatra islands where about 77% of the Indonesian population reside. Using data from Banyumas District, we aimed to investigate the extent of socioeconomic and geographical inequalities in the use, quality, and outcome of the diabetes management program, which represent the general situation regarding this issue in Indonesia.

## Methods

### Study design, population, and setting

This was a cross-sectional study involving 628 participants of the diabetes management program under the Indonesian NHI in Banyumas District, Central Java Province, Indonesia. Banyumas District has an estimated total population of 1.8 million, with an estimated prevalence of diabetes of 1.7%. The NHI covers around 79% of the population, and there are ∼7000 people enrolled in the diabetes management program. The sample size for this study was ∼92.6% of the calculated minimum sample size. Participants were recruited based on the following criteria: (i) participants had been enrolled in the diabetes management program for at least 3 months, and (ii) complete participant data were available for the study.

Participants were consecutively selected from 16 primary care facilities, which provide the first line of diabetes management for participants, between July and October 2021. The 16 primary care facilities were selected from 116 similar facilities in Banyumas District based on the geographical distribution of the participants and the type of primary care facility. Interviewers provided oral explanations about the purpose of the study and the data collection process to the patients who visited the primary care facilities for monthly visits. Patients who agreed to participate in the study signed a written informed consent. The study data were collected by direct interview using a structured data form. Participants who had communication problems (for instance, those who had severe hearing loss) were excluded from the study. All participants had the right to withdraw at any time during the study.

To anticipate a decrease in regular visits of diabetes management program participants to the primary care facilities due to the COVID-19 pandemic, we conducted a telephone survey for participants who did not visit the facilities during data collection. The telephone survey was conducted based on registration data in each primary care facility. For the telephone survey, the interviewer obtained oral informed consent from the participants. This study received ethics approval from the Medical and Health Research Ethics Committee of the Faculty of Medicine, Universitas Jenderal Soedirman, Indonesia (Ref. No. 136/KEPK/VI/2021).

### Variables and measurements

The main outcomes measured in this study were the utilization, quality, and outcome of the diabetes management program. Utilization was measured using regular visits, defined as three consecutive monthly visits by the participants from March to May 2021. Participants of the diabetes program are required to complete a monthly visit from the time of their enrollment in the program to evaluate the treatment progress. The quality of the diabetes management program was indicated by the compliance of the diabetes medication provided by the doctor with the guideline from the Indonesian Endocrinologist Association. The outcome of the diabetes management program was indicated by glycemic control, defined according to the guideline from the Indonesian Endocrinologist Association as an average fasting plasma glucose for three consecutive months of less than 142 mg/dL in the absence of HbA1c level data.

Educational level, employment type, and type of NHI beneficiaries were used as proxies for socioeconomic status. Educational level was defined as the highest level of formal education received by the participants and categorized into the low-educated group, which included participants who never attended school or attended elementary school or junior high school, and the highly-educated group, which consisted of participants who attended senior high school or received higher education.

Employment type was defined as the current employment status of the subjects and categorized into formal workers, consisting of formal employees, self-employed (entrepreneurs and professionals), and pensioners, and informal workers, consisting of subjects who did not receive a regular income (such as daily workers) and unemployed individuals.

The type of NHI beneficiary was categorized into subsidized beneficiaries, including poor and near-poor populations with a government-sponsored NHI premium, and nonsubsidized beneficiaries, that is, NHI beneficiaries whose NHI premium was sponsored by employers, self-funded, or pension-based.

The geographical characteristic was indicated by the place of residence and categorized into urban and rural following the criteria from the Central Statistics Agency.^22^ Data were extracted from the monitoring handbook of the diabetes management program that participants completed during their monthly visits to the primary health care facilities.

### Statistical analyses

Simple inequality measurements were measured in two steps. First, the direct age-sex standardized prevalence rate (SPR) was calculated for each access dimension by groups (e.g., low vs. high educational level). The simple absolute inequality was indicated by the rate difference between the higher group and the lower group. The rate ratio was used to indicate simple relative inequality, which was calculated by dividing the age-sex SPR in the higher group by the age-sex SPR in the lower group.

Initially, sophisticated relative inequality was assessed using multilevel logistic regression, considering the hierarchical structure of the data (nested data). However, results from the initial analysis showed a relatively small variation among clusters, indicating the suitability of using single-level logistic regression. The multiple logistic regression model used dichotomous outcomes (e.g., regular vs. nonregular visits) and socioeconomic status and place of residence as predictors, with the higher groups used as the reference groups. The model was adjusted for demographic characteristics (age and gender), type of primary care facility, and location of the primary care facility. The extent of inequalities was indicated by odds ratios (ORs) and their 95% confidence intervals (95% CIs). Data analysis was conducted using STATA MP 16.0 as the statistical package.

## Results

The basic characteristics of the participants are shown in Table 1. Of the study participants, 76.3% were women and 23.7% were men. The majority of participants were aged 61–70 years old (54.8%), lived in rural locations (78.5%), and were pension-based beneficiaries (59.1%). Most of the primary health care facilities were located in rural areas (65.4%), and most of the participants used a public primary care facility (60.8%). A large proportion of participants had a senior high school level of education (34.1%) and were pensioners (39.5%). In terms of outcomes, 67.2% of participants had monthly regular visits, and 71.8% of participants received standard medication. However, only 35% of participants had good glycemic control.

**Table 1.** Basic characteristics of study participants

Characteristics	***n***	%
Individual		
Gender		
Men	149	23.7
Women	479	76.3
Age, years		
≤50	44	7.0
51–60	159	25.3
61–70	344	54.8
>70	81	12.9
Place of residence		
Rural	493	78.5
Urban	135	21.5
Level of education		
Unschooled	16	2.5
Elementary school	143	22.8
Junior high school	193	30.7
Senior high school	214	34.1
College/university	62	9.9
Employment		
Formal workers	43	6.8
Entrepreneurs/professionals	41	6.6
Informal workers	78	12.4
Pensioners	248	39.5
Unemployed	218	34.7
Type of NHI beneficiaries		
Employer-based	62	9.9
Self-funded	62	9.9
Pension-based	371	59.1
Government subsidized	133	21.1
Primary care facility		
Location		
Rural	411	65.4
Urban	217	34.6
Type		
Independent practitioner	142	22.6
Public primary care	382	60.8
Private clinic	104	16.6
Outcomes		
Regular visits		
Yes	422	67.2
No	206	32.8
Standard medication		
Yes	451	71.8
No	171	21.2
Glycemic control		
Good	220	35.0
Poor	408	65.0

NHI, National Health Insurance.

The main findings of this study are presented in Tables 2 and 3. Using simple inequality measurement (Table 2), socioeconomic inequalities were found for glycemic control. The difference in the prevalence of glycemic control between the highly educated and low-educated groups was 18.4 per 100 participants, and the highly educated group had a ratio of 1.98 for having good glycemic control compared to the low-educated group. Similar results were also found when inequalities were measured based on employment: the difference in the prevalence of glycemic control between formal workers and informal workers was 18.3 per 100 participants, and formal workers had a ratio of 1.97 for having good glycemic control compared to informal workers.

**Table 2.** The extent of simple inequalities in the use, quality, and outcome of the diabetes management program

Inequality dimension	Outcome dimension	SPR (95% CI)^a^		Rate difference	Rate ratio
		High group^b^	Low group^c^		
Level of education	Regular visit	65.1 (61.1–69.1)	83.2 (75.0–91.5)	−18.1	0.78
	Standard medication	69.9 (66.1–73.7)	86.6 (79.1–93.9)	−16.7	0.81
	Glycemic control	37.1 (33.1–41.0)	18.7 (10.0–27.4)	18.4	1.98
Employment	Regular visit	65.1 (61.1–69.0)	83.3 (75.0–91.5)	−18.2	0.78
	Standard medication	69.9 (61.1–73.7)	86.6 (79.2–93.9)	−16.7	0.81
	Glycemic control	37.1 (33.1–41.0)	18.8 (10.0–27.4)	18.3	1.97
Beneficiary type	Regular visit	66.9 (62.8–70.9)	73.1 (65.1–80.9)	−6.2	0.91
	Standard medication	70.8 (66.8–74.7)	80.1 (72.9–87.2)	−9.3	0.88
	Glycemic control	33.6 (29.5–37.7)	45.7 (36.5–54.8)	−12.1	0.73
Place of residence	Regular visit	73.1 (65.3–80.9)	65.3 (61.1–69.4)	7.8	1.12
	Standard medication	85.7 (79.6–91.8)	68.0 (63.9–72.0)	17.7	1.26
	Glycemic control	44.8 (36.0–53.7)	33.1 (29.1–37.1)	11.7	1.35

^a^ SPR with 95% CI, direct standardization with age and sex per 100 participants.

^b^ High group: education>junior high school; employment type: formal workers, entrepreneurs/professionals, pensioners; employer-based, self-funded, pension-based beneficiaries; urban residence.

^c^ Low group: education≤junior high school; employment type: informal workers, unemployed; government-subsidized beneficiaries; rural residence.

95% CI, 95% confidence interval; SPR, standardized prevalence rate.

**Table 3.** The extent of sophisticated inequalities in the use, quality, and outcome of the diabetes management program

Inequality dimension	Outcome dimension		
	Regular visit, OR (95% CI)^a^	Standard medication, OR (95% CI)^a^	Glycemic control, OR (95% CI)^a^
Level of education			
Low	1.00	1.00	1.00
High	1.92 (1.04–3.56)	1.16 (0.59–2.29)	1.91 (0.97–3.76)
Employment			
Informal workers	1.00	1.00	1.00
Formal workers	0.54 (0.20–1.51)	0.97 (0.32–2.88)	1.61 (0.55–4.69)
Beneficiary type			
Subsidized	1.00	1.00	1.00
Nonsubsidized	0.66 (0.36–1.20)	0.89 (0.45–1.73)	0.70 (0.35–1.39)
Place of residence			
Rural	1.00	1.00	1.00
Urban	6.61 (2.90–15.08)	9.73 (3.66–25.90)	3.85 (1.68–8.83)

^a^ Adjusted for age, gender, location of health facility, type of health facility.

OR, odds ratio.

Both absolute and relative geographical inequalities were found, as indicated by the rate difference and rate ratio in the prevalence of regular visits, standard medication, and good glycemic control between urban and rural residents. The difference in regular visits between urban and rural residents was 7.8 per 100 participants, and urban residents were 1.12 times more likely to visit primary care facilities regularly compared to rural residents. The difference in the prevalence rate of standard medication between participants who lived in urban areas and participants who lived in rural areas was 17.7 per 100 participants. The rate of receiving standard medication was 1.26 times higher for urban residents compared to rural residents. Urban residents had a higher prevalence of glycemic control (11.7 per 100 participants) and a rate ratio of 1.35 for having good glycemic control compared to rural residents.

The results of the sophisticated relative inequality measurement are displayed in Table 3. We found mixed results regarding socioeconomic inequalities in the use, quality, and outcome of diabetes management programs. Based on the level of education, participants with higher education had a higher likelihood of making regular visits compared to those with lower education (OR 1.92; 95% CI: 1.04–3.56). For standard medication, the higher education group tended to have a small higher likelihood to receive standard medication compared to the lower education group (OR 1.16; 95% CI: 0.59–2.29), although this finding was statistically insignificant.

Similar findings were found for the outcome of glycemic control (OR 1.91; 95% CI: 0.97–3.76). Based on employment status, we found that formal workers tended to have a lower likelihood to have regular visits (OR 0.54; 95% CI: 0.20–1.51) and receive standard medication (OR 0.97; 95% CI: 0.32–2.88) compared to the informal workers although these findings were statistically insignificant. For the glycemic control, a higher likelihood of better glycemic control was found in formal workers (OR 1.61; 95% CI: 0.55–4.69) compared to informal workers although this finding was also statistically insignificant.

Based on the type of NHI beneficiaries, we found that for all three outcomes, nonsubsidized beneficiaries had a lower likelihood to have regular visits (OR 0.66; 95% CI 0.36–1.20), received standard medication (OR 0.89; 95% CI: 0.45–1.73), and have better glycemic control (OR 0.70; 95% CI: 0.35–1.39) compared to the subsidized beneficiaries although all these results were statistically insignificant. Contrary to findings regarding socioeconomic inequalities, we found geographical inequalities to a relatively large extent for all three outcomes of the diabetes management programs. Participants who lived in urban areas had a higher likelihood of having regular visits (OR 6.61; 95% CI: 2.90–15.08), receiving standard medication (OR 9.73; 95% CI: 3.66–25.90), and having good glycemic control (OR 3.85; 95% CI: 1.63–9.93) compared to those who lived in rural areas.

## Discussion

This study aimed to analyze the extent of socioeconomic and geographical inequalities in the use, quality, and outcome of diabetes management programs among NHI beneficiaries in Indonesia as indicated by regular visits, standard medication, and glycemic control. Findings from our study showed that most of the participants had poor diabetes outcomes as indicated by the large proportion of individuals with poor glycemic control. Educational inequalities were found in the use of the diabetes management program among the Indonesian NHI beneficiaries. Relatively large geographical inequalities were also found, with urban residents having more regular visits, receiving more standard medication, and having better glycemic control compared to rural residents. Unexpectedly, the tendencies of reverse inequalities were found for socioeconomic inequalities which were estimated based on employment status and type of NHI beneficiaries.

To our knowledge, this study was the first to assess the inequalities in the use, quality, and outcome of a diabetes management program under NHI in the context of a LMIC. Using primary data collected from a representative sample of a relatively large-scale diabetes management program in Indonesia, this study provides useful insights into the impact of the NHI program on the use, quality, and outcome of diabetes management programs for participants with heterogeneous backgrounds.

However, this study is not without limitations. The data collection was conducted right after the second wave of the COVID-19 pandemic in Indonesia, which likely reduced the number of participants who visited the primary care facilities. This may have led to the possibility of selection bias when the data were collected only from participants who were regular visitors to the primary care facilities. Ideally, this study used registry-based data from the Indonesian NHI database. However, since the NHI office did not grant access to the NHI individual database, this study had to use primary data collected from the participants. To minimize the possibility of selection bias, we used secondary data from the registration system in each primary care facility to validate the primary data and to include participants who did not visit the primary care facility.

The findings from this study indicate that the utilization of the diabetes management program and standard medication is relatively adequate and comparable to those from studies in high-income countries and other LMICs.^3,23^ The relatively high utilization of the diabetes management program among Indonesian NHI beneficiaries may be due to several factors. First, health care utilization in this study was measured among the population covered by the NHI program, while previous studies have mostly used the general population. Health insurance is likely to remove most financial barriers to accessing the disease management program, resulting in higher utilization compared to the general population.^24^

Second, the diabetes management program is provided by primary care facilities, which are adequately numbered and evenly distributed. Public primary care facilities are available at the subdistrict level and are accessible to participants.^25,26^ Third, public knowledge, particularly among NHI beneficiaries, is improving and is likely higher than in the general population, which may increase the use of the diabetes management program.^27^

Although the utilization and standard medication among participants in the diabetes management program under the Indonesian NHI are relatively adequate, the outcome of the program falls far short of global figures. Studies show that globally, ∼50% of participants in diabetes management programs achieve good glycemic control.^24,28^ Glycemic control in diabetes depends on various factors beyond medical treatment, such as behavior, environment, and culture.^29^ In the Indonesian context, the culture of consuming foods high in sugar in some ethnic groups is an example of how culture can impact disease control.^30^

This study shows that there are socioeconomic, particularly education-related, inequalities in the use of the diabetes management program among Indonesian NHI beneficiaries. This finding is similar to previous studies conducted in LMICs among the general population.^18^ The material resource hypothesis may explain this finding. For example, highly educated individuals tend to have more financial stability, which plays an important role in disease control. Financial stability allows individuals to manage their time to have regular visits to health facilities and adjust their lifestyles (such as through dietary changes and regular exercise), which are beneficial for disease control.^31^ Contrary, low-educated people have the limitation to implement the planned treatment such as regular visits and lifestyle changes due to the economic constraint.^32^

Another mechanism that may explain this finding is related to the indirect costs of accessing health care. While the benefits of NHI only cover the medical costs of the diabetes management program, participants still have to bear indirect costs when using health care, such as transportation costs and opportunity costs. Opportunity costs arise when participants have to allocate their working time to visit a health care facility.^17,25^

Highly educated people, who usually have regular working hours and income, may have less of a trade-off in allocating time to regularly visit health care facilities compared to low-educated people. In contrast, low educated people have limited options to allocate time for regular health care visits due to their reliance on daily income to meet basic needs, which would be compromised by attending health care facilities.^33^ In addition, highly educated people may be more likely to have positive prior health care experiences positively impacting trust in health authorities, batter access to reliable health information sources, and better understand the perceived benefits of regular visits to the diabetes management program compared to the low educated people.^14,16^

This study highlights the significant geographical inequalities in the use, quality, and outcome of diabetes management programs among Indonesian NHI beneficiaries. Geographical inequalities in accessing health care are a common phenomenon in Indonesia.^17,25^ Rural communities often have low health literacy, which leads to a lack of understanding of the disease and low awareness of the need to seek health care. Rural areas also have a large variety of landscapes that can cause transportation barriers when people need to visit health care facilities, such as higher travel costs and longer travel times.^17^

In addition, the large disparity in the availability of services between urban and rural areas is likely to cause significant access problems. The number of health care facilities in rural areas is much lower compared to urban areas, limiting the options for participants living in rural areas. The resource disparities in terms of personnel, medical equipment, drugs, and infrastructure between urban and rural areas may cause inequalities in treatment compliance with guidelines, leading to inequalities in glycemic control.^34^ Previous studies have shown significant disparities in health resources between regions in both Indonesia and other LMICs.^35^

Findings from our studies reveal the tendency of reverse socioeconomic inequalities in use, quality, and outcome of diabetes management program for several estimates particularly for the estimates based on type of NHI beneficiaries. Previous studies show that the association between socioeconomic status, particularly employment status, and health insurance status in low-middle income countries is still inconclusive.^36^

In the context of Indonesian NHI, nonsubsidized beneficiaries consist of formal workers with most of them being blue collar/low-skilled manual workers and small entrepreneurs. Arguably, this population group has low job control which may be related to the lower probability to have regular visit, receive standard medication, and have better glycemic control. Low job control causes inflexible working time which creates problems visiting the primary health care facility regularly, particularly when the programs are only available during office hours and on one specific date every month.^37^ Irregular visits are likely to cause lower quality of treatment since the treatment needs to be adjusted regularly based on clinical condition of the participants. This may become an important barrier to achieve better glycemic control. In addition, low-skilled manual workers tend to have unhealthy diet pattern, limited physical activity, and higher working stress, which have been attributed to worse glycemic control.^38,39^

## Conclusions

The implementation of diabetes management programs for the beneficiaries of Indonesian NHI may improve people's access to the services by removing the direct medical cost. However, substantial socioeconomic and particularly geographical inequalities in the use, quality, and outcome of diabetes management programs were still observed among participants. This suggests the need for further efforts to create more equal access to these programs, which goes beyond simply removing medical cost barriers.

Adjustments, such as providing more flexible office hours, offering mobile services, and conducting community engagement and education programs (e.g., public health campaigns and culturally tailored community education) to bring the program closer to the participants, can be considered to improve accessibility. Reducing geographical disparities in availability of skilled health personnel in diabetes management and available medical equipment should be a priority in the long-term strategy to improve access to diabetes management programs under the Indonesian NHI. Findings from this study underline the importance of addressing inequalities in the use, quality, and outcome of diabetes management programs as part of pivotal strategies to reduce the inequity in diabetes prevalence, as well as the overall health inequity, in Indonesia.

## Abbreviations Used

95% CI: 95% confidence interval
LMIC: low-middle-income country
NCD: noncommunicable disease
NHI: National Health Insurance
OR: odds ratio
SPR: standardized prevalence rate
